# Recent Advances in the Management of Breathlessness

**DOI:** 10.4103/0973-1075.76238

**Published:** 2011-01

**Authors:** Katrina Breaden

**Affiliations:** Flinders University, Adelaide, Australia

**Keywords:** Refractory breathlessness, Supportive care, Palliative care

## Abstract

Breathlessness is a frightening symptom to both witness and experience. It is common in many conditions, especially in the palliative setting, profoundly affecting the quality of the person’s life. The purpose of this article is to provide an overview of the recent advances in the management of breathlessness in the areas of, knowledge of disease trajectories, assessment, pharmacological and non-pharmacological interventions and the use of oxygen.

## INTRODUCTION

*Breathlessness is a complex experience of the mind and the body.*[1]

Expanding on the above quote, the sensation of breathlessness involves not just the body and mind, but the spirit as well. It is a frightening symptom to both witness and experience and is common in many conditions, especially in the palliative setting, profoundly affecting the quality of the person’s life. Every activity, from showering to eating is an enormous struggle, leading to a contracted world for the sufferer and his or her family. Anxiety and fear are constant companions.[2]

Breathlessness has been variably defined as an unpleasant awareness of breathing, or an uncomfortable sensation.[3] There are many causes of breathlessness, too numerous to mention here, but they all fall into one of two categories; cancer related or noncancer related. The incidence of breathlessness ranges from 21% to 90% in cancer patients, depending on the severity and type of cancer involved.[4] In those people who suffer from heart failure or lung disease, the incidence is approximately 65% and 90%, respectively.[5] Breathlessness occurs nearly universally as death approaches.[6]

The purpose of this paper is to provide an overview of the recent advances in the management of breathlessness. Before outlining these interventions, it is helpful to examine aspects of the breathlessness trajectory as it relates to the type of disease involved.

## DIFFERENT TRAJECTORIES

There is a difference in the trajectories of breathlessness in terms of prevalence and severity depending on the type of diagnosis. People with chronic obstructive pulmonary disease (COPD) often have slowly developing breathlessness over a number of years made worse by exertion until it is present even at rest. The slow decline is interspersed with exacerbations of severe breathlessness which may require admission to hospital; death may occur unpredictably.[7] In the final stages of this disease, breathlessness is the most frequently reported symptom.[8] One large Australian study (*n* = 5862) exploring the perception of breathlessness in the last three months of life found that this symptom was more severe in people with a non-cancer diagnosis (mainly COPD) and the level of severity was constant over the three-month period prior to death despite treatment. In contrast, people with cancer had initially lower levels of breathlessness than the non cancer group, but this level increased during the last 10 days of life.[9] In addition, in the cancer setting, the presence of breathlessness indicated a shorter prognosis and often developed rapidly[10] This information has implication for resource allocation and service planning-if a palliative service only admits those patients with cancer, then it is missing out on seeing many people with distressing levels of breathlessness. People with nonmalignant or malignant associated breathlessness have similar level of need.

## ASSESSMENT

During the assessment of breathlessness, it is important to ask why-why this symptom at this time? The Flinders University teaching team has named this clinical decision and assessment framework, the “Why Framework” giving priority to the possibility of potentially reversible symptoms. Within this framework, there are two important guiding questions:

1. Is this an expected or unexpected problem? and,
2. Can (and should) we do something about it? This decision making requires an understanding and knowledge of the disease trajectory, differential diagnoses and reversible and nonreversible causes.[11]

### Assessment tools

Physiological indicators such as arterial blood gases, oxygen saturation, spirometry or respiratory rate do not necessarily correlate with the degree of breathlessness; they are not direct measures.[7,12] Therefore, other assessment methods are required. As it is a subjective experience, patient self-report is vital in assessment-not simply staff report. A visual analog scale is well suited for measuring the intensity of the symptom[13] as is the Borg scale.[14] Alternatively, a quality of life measurement might be more appropriate for an holistic assessment, including the impact that the breathlessness has on a person’s physical, emotional, and social functioning.[7]

In spite of our best efforts to manage the reversible causes, for many people breathlessness remains to exist. Breathless is then considered to have become intractable, i.e., It has become *refractory*.[15]

### Recent advances in the management of refractory breathlessness

The treatment of breathlessness is complex. It depends on the underlying causes and the potential to reverse the reversible. Management requires, if possible, a multidisciplinary team involvement and needs to focus on the following:[16]

1. Address and relieve discomfort and distress of patient and their carers; and
2. Reverse or treat the disease process if possible, and appropriate.

In order to accomplish these aims, current evidence highlights the following pharmacological and nonpharmacological strategies in the management of refractory breathlessness.

### Pharmacological

- Opioids-either oral or parenteral-are now considered to be the gold standard in reducing ventilatory demand.[17] A slow release preparation of morphine has been found to be beneficial.[15] The opioid dose will depend on whether the patient is opioid-naive or not. The patient subpopulation that will benefit most from this intervention is still being investigated.[18] There is little or no evidence for the use of nebulized opioids in the management of breathlessness.[19] However, inhaled frusemide has shown promise in some preliminary studies.[20]
- Anxiolytics may assist in the anxiety component of breathlessness. However, these may be poorly tolerated in some patients, especially in those with liver failure.[21] Benzodiazepines such as oxazepam have a shorter half-life and, therefore, less sedating effects than some other preparations.[22] However, newer evidence from a recent Cochrane review has found that benzodiazepines did not improve breathlessness in people with COPD or advanced cancer, yet they may have a role to play when other means have failed.[22] The use of sedatives in the management of breathlessness requires careful consideration and more research is needed in this area.[21] Phenothiazines may also be useful in treating anxiety associated with breathlessness.[23]
- Long acting beta agonists may be beneficial in breathlessness due to COPD in reducing the work of breathing.[7,26] Bronchodilators help in relaxing muscles and improving muscle tone in the airways. The correct technique and the use of a spacer are vital to ensure that the full dose reaches the airways.[23]

### The use of oxygen

- The use of oxygen has been shown to be no better than room air administered through nasal cannulae at 2 l/min in patients (especially in people with cancer) who are mildly or non-hypoxic, i.e., paO _2_<55 mgHg.[24,25]

A trial of 3–4 days should be sufficient to show whether there will be a benefit or not in its application.

### Nonpharmacological management

The nonpharmacological methods outlined below will not suit every patient. The key is to tailor interventions to the individual so that they are congruent with their values and beliefs concerning health and illness. Many of the following strategies can be nurse-led.

- Listen to the patients’ experience and avoid telling them to just “calm down”.[1]
- Fans/open windows/cold washers on the face are often helpful in reducing the sensation of breathlessness. The effect is thought to be due to the stimulation of the second and third branches of the trigeminal nerve.[1]
- There does seem to be a relationship between anxiety and breathlessness, however, which one comes first is difficult to tell. Strategies such as relaxation training and distraction do seem to help as do cognitive behavioral therapies. However, such strategies need to be introduced early in the disease trajectory as their introduction in the last days of life is seldom appropriate when significant fatigue may also be present.[1]
- The effectiveness of physical conditioning has been shown to be useful in COPD patients, and in those with cancer.[2] We also know that taking to one’s bed results in a worsening of breathlessness.
- Acupuncture may be beneficial in people with COPD. There has been some preliminary research into this therapy. Lung function and exercise tolerance does seem to improve after acupuncture.[27,28] The reduction in breathlessness is thought to be due to the release of endogenous opioids.[29] More research is needed in this area.
- Advanced planning in the event of an acute exacerbation is vital and conversations regarding where the person wants to be cared for in such an event needs to be documented.[30]
- Controlled breathing exercises and techniques such as an upright leaning forward position and pursed lip breathing are also beneficial.[21]
- Chest wall vibration, neuroelectrical muscle stimulation, walking aides, and breathing training were found to be effective in reducing breathlessness in a recently conducted systematic review.[31]

Many of the above strategies are not necessarily new, yet research evidenced is building in several of these areas to underpin what has been used for decades in the management of refractory breathlessness. It does seem, however, that psychological support, breathing exercises and the development of coping strategies can assist patients in the management of refractory breathlessness.[32]

## CONCLUSION

Breathlessness is a common symptom in palliative care and its causes are multiple. It is a complex symptom to manage and needs the input from a multidisciplinary team. There is growing evidence for the use of both pharmacological and nonpharmacological interventions; however, further research is needed to firmly establish the best way forward in treating refractory breathlessness.
